# Adolescent socio-economic and school-based social status, health and well-being

**DOI:** 10.1016/j.socscimed.2014.09.037

**Published:** 2014-11

**Authors:** Helen Sweeting, Kate Hunt

**Affiliations:** MRC/CSO Social and Public Health Sciences Unit, University of Glasgow, 200, Renfield Street, Glasgow G2 3QB, UK

**Keywords:** Adolescent, Subjective social status, Socio-economic status, School-based social status, Peer status, Health, Psychological well-being, United Kingdom

## Abstract

Studies of adults and adolescents suggest subjective socio-economic status (SES) is associated with health/well-being even after adjustment for objective SES. In adolescence, objective SES may have weaker relationships with health/well-being than at other life stages; school-based social status may be of greater relevance. We investigated the associations which objective SES (residential deprivation and family affluence), subjective SES and three school-based subjective social status dimensions (“SSS-peer”, “SSS-scholastic” and “SSS-sports”) had with physical symptoms, psychological distress and anger among 2503 Scottish 13–15 year-olds. Associations between objective SES and health/well-being were weak and inconsistent. *Lower subjective SES* was associated with increased physical symptoms and psychological distress, *lower SSS-peer* with increased psychological distress but reduced anger, *lower SSS-scholastic* with increased physical symptoms, psychological distress and anger, and *lower SSS-sports* with increased physical symptoms and psychological distress. Associations did not differ by gender. Objective and subjective SES had weaker associations with health/well-being than did school-based SSS dimensions. These findings underline the importance of school-based SSS in adolescence, and the need for future studies to include a range of school-based SSS dimensions and several health/well-being measures. They also highlight the need for a focus on school-based social status among those working to promote adolescent health/well-being.

## Highlights

• We examined associations between adolescent social status and several health measures.
• Models included objective and subjective socio-economic status and school peer, scholastic and sports status.
• School status had stronger associations with health than did socio-economic status.
• Different school status dimensions and health measures were associated in different ways.
• Studies should include a range of school status dimensions and health measures.

## 1 Introduction

As many have observed, social status has both material and psychosocial dimensions (Marmot, 2005) and operates on different structural levels (Almquist, 2009). In adolescence, school-based social status may be particularly important (Goodman et al., 2001, Karvonen and Rahkonen, 2011). This paper examines associations between several measures of socio-economic and school-based status and adolescent health/well-being.

Innumerable studies have found higher *objective* socio-economic status (SES) to be associated with better health/well-being among younger children and adults (Marmot, 2005). Some studies of adolescents report similar findings (Goodman et al., 2007, Goodman et al., 2005, Koivusilta et al., 2006, Richter et al., 2012). However, others have found little or no evidence of consistent differentials in (self-report) health/well-being measures according to various indicators of parent or household SES or area-based deprivation (Fagg et al., 2013, Glendinning et al., 1992, Spencer, 2006).

Subjective social status (SSS) has been defined as a person's sense of place within a hierarchy, which may or may not reflect objective status (Adler and Stewart, 2007). There is evidence that measures of SSS are associated with health/well-being even after adjustment for ‘objective’ SES in both adults (Adler et al., 2000, Singh-Manoux et al., 2003) and adolescents (see below). SSS may summarise material and social circumstances better than ‘objective’ measures, partly because it allows individuals to incorporate past achievements and future prospects into their overall self-placement (Singh-Manoux et al., 2003), thus representing the varied experiences of those categorised as equivalent via ‘objective’ SES measures (Goodman et al., 2007).

To measure SSS, the most commonly used (MacArthur) scale asks respondents to mark where they would place themselves on a picture of a ladder (Adler and Stewart, 2007). SSS scales for adolescents have asked respondents to indicate where “your family would be on this ladder” (‘SSS-society’) and, on the basis that school is their most salient community, where they would place themselves on a ladder where the highest rung represents “the people in your school with the most respect, the highest grades and the highest standing” (‘SSS-school’) (Goodman et al., 2001).

While SSS-society represents macro-level status measures, SSS-school reflects more immediate, micro-level processes (Almquist, 2009) which might be of greater relevance to adolescents (Goodman et al., 2003, Karvonen and Rahkonen, 2011). This would suggest that in analyses including both measures, gradients in adolescent self-report health/well-being should be stronger in respect of SSS-school than SSS-society. Three general population studies have examined this, with contrasting results. SSS-school showed stronger associations with health/well-being (depressive symptoms and obesity) among US 13–16 year-olds (Goodman et al., 2001) and measures of sleep in Canadian 11–17 year-olds (Jarrin et al., 2014). However, among Central and Eastern European 15–17 year-olds, SSS-society had a stronger relationship with self-rated health than did SSS-school (Page et al., 2009). An Australian study of 11–19 year-olds with refugee backgrounds which included SSS-school and two SSS-society ladders (relating to the ethnic and the broader Australian communities) found SSS in the broader Australian community (described by the authors as indicating “belonging”) was the strongest predictor of health and well-being (Correa-Velez et al., 2010). Another study, of Swedish 15–18 year-olds, found “attributed status” (summed ladders representing family wealth and position in society) and “acquired status” (ladders representing social position in relation to friendship group and to schoolmates) each had similar associations with depression (Aslund et al., 2009).

Other studies of adolescents have included one SSS ladder measure only. Most have focused on SSS-society, with mixed findings. Thus, following adjustment for objective SES, lower SSS-society was associated with poorer self-rated health, both cross-sectionally and longitudinally in US 12–19 year-olds (Goodman et al., 2007), with poorer self-rated health, increased health complaints, chronic illness and psychological distress in Finnish 15 year-olds (Karvonen and Rahkonen, 2011) and with presence of mood, anxiety, disruptive behaviour and substance disorders in US 13–18 year-olds (McLaughlin et al., 2012). However, in other studies, SSS-society was associated with health (stress) only among certain sub-groups of US 12–19 year-olds (Goodman et al., 2005) or with certain health measures (positive psychological characteristics) but not others (physiological measures; negative psychological characteristics) among US 14–19 year-olds (Chen and Paterson, 2006). Significantly, none of these studies were conducted in the UK, a country characterised by large socio-economic inequalities compared with other industrialised nations (Hills, 2010). There has been much less research interest in SSS-school. However, a study of US 12–18 year-old females (which did not measure SSS-society) found low SSS-school was associated with subsequent BMI increases (Lemeshow et al., 2008).

Another layer of complexity in respect of SSS-school is that this measure reflects various status dimensions (Finkelstein et al., 2006, Wilkinson et al., 2009). Indeed the author of the first study to include the measure notes that “the three anchoring constructs, respect, standing, and grades, are not necessarily well correlated, nor are they intended to measure the same thing” (Goodman et al., 2003) p. 1019. This reflects the multidimensional nature of school-based status; high status might result because an adolescent is liked by others, highly visible or powerful and/or from achievements in academic, sporting or other school-defined goals (Sweeting et al., 2011).

Research into associations between different dimensions of school-based status and health/well-being is largely located within the developmental psychology, rather than health inequalities, literature. The focus has therefore been on psychological distress and/or self-esteem. Most studies using sociometric techniques to measure classroom popularity (pupils nominate others as liked, disliked, a friend, etc), have found associations between this measure of status and health/well-being, particularly psychological distress (Kiesner, 2002, Sandstrom et al., 2003). Self-esteem is higher and depression lower among peer-rated members of high status US adolescent crowds (“populars”, “jocks”), compared with low status crowd members (Brown et al., 2008). A meta-analysis concluded that associations between children's and adolescents' social status and negative emotionality tend to be moderate-to-small, while those with positive emotionality tend to be small (Dougherty, 2006). Focusing on relationships between adolescent health/well-being and status within officially sanctioned school-defined hierarchies, studies generally find better self-rated health and psychological well-being among those with higher academic interest and achievement (Delsing et al., 2007, Koivusilta et al., 2006) and, unsurprisingly, those engaging in more physical activity and/or sports team participation (Johnson and Taliaferro, 2011).

Such studies suggest high status, regardless of dimension, is associated with better health/well-being. However, it has been suggested that in adolescence, aspects of popularity may “prove stressful or taxing over time” (Schwartz and Gorman, 2011) (p. 249). Qualitative studies have highlighted pressures associated with maintaining high popularity among adolescent females (Eder, 1985, Michell, 1997). Consistent with this, a study employing identical school-based status measures to those in this paper, found lower cortisol levels among 15 year-olds with high academic and sports status, but higher levels among those with high peer status (West et al., 2010).

Another potentially important issue for studies of school-based status and health/well-being is “the two cultures of boys and girls” (Rose et al., 2011). While adolescent females tend to closer relationships, sensitivity to others and generally more positive learning attitudes, males often place more value on sports-related achievements (Giordano, 2003, Michell, 1997). Associations between both classroom popularity and academic achievement and health/well-being may therefore be stronger for females, while those between sports achievement and health/well-being may be stronger for males. Evidence on this is sparse. However among Dutch adolescents, depressive problems were most strongly associated with not being liked among females, but with not being good at sports among males (Oldehinkel et al., 2007), while among Hungarian students, physical activity had a much stronger association with self-rated health among males than females (Piko, 2000). A longitudinal study of US children found reductions in anxiety/depression among females were predicted by having previously been nominated by other pupils as liked, but by having previously been nominated as popular among males (Sandstrom and Cillessen, 2006). Another study found relationships between childhood peer status and subsequent adult anxiety and depression in females but not males (Modin et al., 2011).

### 1.1 The current study

In this paper we analyse data from Scottish adolescents to identify the relative importance of objective social status, SSS-society (henceforth ‘subjective SES’) and SSS-school for adolescent health/well-being in the UK. We also address three sub-questions raised by the literature: (1) does the relative importance of different status measures vary across different health/well-being measures; (2) are different dimensions of SSS-school associated with health/well-being in the same way; and (3) are there gender differences in associations between SSS-school dimensions and health/well-being?

Our two ‘objective’ SES measures comprise residential area deprivation and family affluence; the subjective SES measure is the SSS-society ladder. The SSS-school dimensions relate to adolescent ‘peer’, ‘scholastic’ and ‘sports’ status (described below). Similar previous studies have included a range of health/well-being measures, principally self-rated health, psychological distress and self-esteem. Our health/well-being measures include physical symptoms, psychological distress and anger. We include physical symptoms because (developmental psychology) analyses of associations with dimensions of school-based status have focused on psychological measures. Associations between SSS-school (in particular) and physical symptoms may suggest micro-level processes associated with low SSS influence health by getting “under the skin” and impacting on physiology (Berkman et al., 2000, Hertzman and Boyce, 2010). Anger is of interest given its associations with mental health problems, problem behaviours, poorer self-report health and elevated blood pressure and heart rate in adolescence, and with higher levels of both psychological and physical morbidity and mortality in adulthood (Kerr and Schneider, 2008, Novin et al., 2010).

In addition to addressing the questions above, and since, so far as we are aware, this is the first UK study of adolescents to use the SSS-society ladder, we also examine associations between our objective and subjective SES measures, and compare results from our sample with those from both the US (Chen and Paterson, 2006, Goodman et al., 2003, Goodman et al., 2001, Goodman et al., 2007, Goodman et al., 2005) and Finland (Karvonen and Rahkonen, 2011).

## 2 Methods

### 2.1 Sample

Data were obtained from Scottish Secondary school pupils, first surveyed in 2010 and followed up in 2011. The study was approved by the relevant Glasgow University Ethics Committee, local education authorities and schools.

To maximise representativeness we selected seven schools with different socio-economic catchments (indicated by proportions receiving free school meals) from two urban and semi-rural areas in Scotland's central belt. At both baseline and follow-up, all pupils in selected year groups were invited to participate via letters to parents including parental opt-out consent forms. Pupils separately received study information and gave written consent prior to participation. Levels of non-consent were very low; almost all non-responders were those absent on the survey days. The baseline sample comprised 2937 pupils in Scottish Secondary 1–3 (S1–S3) year groups (92% of the eligible sample of 3189), of whom 2503 also participated in 2011, when they were in the S2–S4 year groups (aged 13–15). The analyses reported here use data obtained in 2011, apart from one measure (family affluence), which was only reported in 2010, thus restricting the sample to those participating at both dates. (Residential deprivation and subjective SES measures were not included in 2010, thus precluding longitudinal analyses including all variables at both baseline and follow-up.)

Pupils completed questionnaires in examination conditions during school-based sessions, led by researchers and survey assistants, with a ratio of around one to every ten pupils. Teachers, if present, were strongly encouraged not to intervene, and none had access to completed questionnaires.

To ensure confidentiality, but also allow linkage of data from both dates, the baseline and follow-up questionnaires had identical front sheets. Pupils wrote their name, birth date, mother's and father's first names, then transferred pre-assigned letters/digits from these answers onto the main questionnaire. A survey team member checked this transfer, removed the front sheet and tore it up in view of the pupil. This procedure has been successfully used previously (Galanti et al., 2007).

### 2.2 Measures

All measures apart from family affluence were obtained at the 2011 survey.

#### 2.2.1 Residential deprivation

Represented via the Scottish Index of Multiple Deprivation (SIMD-2009). This identifies concentrations of multiple deprivation across Scotland by assigning a score to small areas (median population around 750), derived from national indicators covering seven domains (income, employment, health, education, access to services, housing, crime). The methodology used to construct the SIMD is widely accepted; similar methodologies are used across Great Britain and Northern Ireland (Scottish Government, 2009). SIMD deciles are ranked 1 (most deprived) to 10 (least deprived) and were available for 77% of our analysis sample.

#### 2.2.2 Family affluence scale (FAS)

This scale asks about number of family cars, vans/trucks; family computers; past year family holidays; and own (not shared) bedroom. It has been found to be reliable (pupils' and parents' reports on component items agree) and sensitive in differentiating between-country levels of affluence (Currie et al., 2008). Scores range from 0 to 7.

#### 2.2.3 Subjective SES

The youth version of the MacArthur Scale of Subjective Social Status (Goodman et al., 2001) was used, with wording adapted for Scottish adolescents. The questionnaire included an image of a 10-rung ladder with the instructions: “Imagine that this ladder shows how Scottish society is set up. Now think about your family. Please tell us where you think your family would be on this ladder”. The top rung was labelled “the best off people in Scotland – they have the most money, the most education, and the jobs that bring most respect” and the bottom rung “the worst-off people in Scotland – they have the least money, not much education and no job, or a job that no-one wants or respects”. Pupils were instructed to “Put a cross which shows best where your family would be”.

#### 2.2.4 SSS-school dimensions

Based on previous work (Sweeting et al., 2011), the questionnaire included seven pictures of a 10-rung ladder, with the instructions “Imagine these ladders show where people fit in your year group. Where would you put yourself?”. Pupils used these ladders to rate themselves on: popularity, doing well at school, being powerful, a trouble-maker, attractive or stylish, respected, and sporty, compared with the rest of the year group. As previously (Sweeting et al., 2011) factor analysis suggested three dimensions, described here as “SSS-peer”, “SSS-scholastic” and “SSS-sports” (details below).

#### 2.2.5 Health/well-being measures

The questionnaire asked pupils whether they had suffered from each of 11 symptoms in the past month. We classified seven as ***physical symptoms*** (headaches; asthma or wheeze; sickness or stomach aches; fainting; aches; colds or flu; skin problems). We have adopted this categorisation in several previous analyses (e.g. Sweeting and West, 2003), while remaining aware of debates over the classification of symptoms as ‘physical’ or ‘malaise’ (Popay et al., 1993). Pupils reporting five or more (29.2%) were categorised as reporting ‘high’ physical symptoms. To measure psychological distress, pupils completed the ***12-item General Health Questionnaire (GHQ-12)*** which has been validated for use with younger adolescents (Tait et al., 2003). The GHQ was designed as a measure of state, focusing on inability to carry out normal functions and emergence of distressing symptoms. Each item includes four answer options. We used binary scoring (0-0-1-1) and the standard cut-off of 2/3 (Goldberg and Williams, 1988). This classified 24.2% as GHQ ‘cases’ (potentially clinically significant levels of distress). To represent ***anger***, responses to a single item (‘I get angry when anybody tells me what to do’) were dichotomised into ‘describes me very/quite well’ (19.4%) and ‘describes me a bit/not at all’.

### 2.3 Analyses

Analyses were conducted using Stata 11.1. First, data reduction in respect of the school-based ladder scores using principal components analysis with orthogonal rotation suggested a three factor solution (Supplementary Table 1). The factors were labelled “SSS-peer” (including “popular”; “powerful”; “respected”; “attractive or stylish”; “trouble-maker”), “SSS-scholastic” (“doing well at school”; not a “trouble-maker”) and “SSS-sports” (“sporty”). Correlations determined associations between the objective and subjective SES measures. One-way ANOVA identified gender and year group differences in subjective SES.

Bivariate and mutually adjusted associations between each status measure and health/well-being were determined via logistic regression. To investigate whether both high *and* low status were associated with poorer health/well-being, each status measure was collapsed into three categories representing (approximately), the lowest 25%, mid 50% and highest 25% of the sample, with an additional ‘missing’ category for residential deprivation (results relating to this not discussed further). Tests of interactions with gender were conducted to identify any gender differences in the status-health/well-being associations. Since none were identified (further details below), analyses of associations between status and health/well-being were conducted on the whole sample, with the mutually adjusted analyses also adjusting for gender and year group.

Comparison of our baseline sample with a Scotland-wide school-based survey, conducted at the same time, showed similar family affluence levels (Sweeting et al., 2012). Probabilistic weights have been derived to compensate for differential attrition at follow-up. The data were also clustered within school classes. Since almost identical results were obtained in analyses based on weighted data, accounting for clustering (Supplementary Table 2) and those which did neither, results are presented on unweighted data, without accounting for clustering. Analyses of associations between status and health/well-being were conducted on those with complete data on all relevant variables (*N* = 2313 for physical symptoms; 2304 for GHQ; 2300 for anger).

## 3 Results

Mean subjective SES in our sample was 6.64 (SD = 1.50); proportions reporting ‘low’, ‘average’ and ‘high’ levels (ladder rungs 1–3, 4–7 and 8–10) were 2.5%, 75% and 27.5%. Subjective SES was higher among males (6.71) than females (6.58) (*p* = 0.039) and among younger pupils (means of 6.83 in the S2 year group, 6.68 in S3 and 6.40 in S4; *p* < 0.001). Relationships between our objective and subjective SES measures were all weak, although significant due to large sample size (residential deprivation with family affluence *r* = 0.292, (*p* < 0.001); residential deprivation with subjective SES *r* = 0.084, (*p* < 0.001); family affluence with subjective SES *r* = 0.208, (*p* < 0.001)).

Table 1, Table 2, Table 3 show associations which each health/well-being measure had with gender, year group and all status measures (unadjusted and adjusted). Because we were interested in whether there were gender differences in associations between the three SSS-school dimensions and health/well-being, we began by examining interactions with gender (Supplementary Table 3). None of the SSS-school and health/wellbeing associations differed significantly between males and females. Analyses of these associations were therefore conducted on the whole sample.

**Table 1:** ‘High’ physical symptoms according to gender, school year group and status measures: (a) numbers (and row percentages); (b) unadjusted odds ratios (and 95% confidence intervals) showing bivariate associations with gender, year group and each status measure; (c) fully adjusted ORs (and 95% CIs) for model including gender, year group and all status measures.

	(a) Total symptoms				(b) High symptoms		(c) High symptoms	
	‘Low’		‘High’					
	*N*	row %	*N*	row %	OR (95% CI)	Wald	AOR (95% CI)	Wald
**Gender**								
Males	833	70.7	345	29.3	1.00		1.00	
Females	680	59.9	455	40.1	1.62 (1.36–1.92)***	5.4	1.56 (1.30–1.88)***	4.7
**Year group**								
S2	537	67.6	258	32.5	1.00		1.00	
S3	508	65.8	264	34.2	1.08 (0.88–1.33)	0.7	1.06 (0.85–1.31)	0.5
S4	468	62.7	278	37.3	1.24 (1.00–1.53)*	2.0	1.16 (0.94–1.44)	1.4
**Residential deprivation**								
High status (low deprivation)	384	68.6	176	31.4	1.00		1.00	
Medium status	577	64.2	322	35.8	1.22 (0.97–1.52)	1.7	1.17 (0.93–1.48)	1.4
Low status (high deprivation)	219	65.4	116	34.6	1.16 (0.87–1.54)	1.0	1.06 (0.79–1.44)	0.4
Missing	333	64.2	186	35.8	1.22 (0.95–1.57)	1.5	1.17 (0.90–1.52)	1.2
**Family affluence scale**								
High status (high affluence)	356	61.3	225	38.7	1.00		1.00	
Medium status	805	66.8	401	33.3	0.79 (0.64–0.97)*	−2.3	0.76 (0.61–0.94)*	−2.5
Low status (low affluence)	352	66.9	174	33.1	0.78 (0.61–1.00)	−2.0	0.67 (0.51–0.87)**	−3.0
**Subjective SES**								
High status	436	68.6	200	31.5	1.00		1.00	
Medium status	806	66.1	413	33.9	1.12 (0.91–1.37)	1.1	1.11 (0.89–1.37)	0.9
Low status	271	59.2	187	40.8	1.50 (1.17–1.93)**	3.2	1.44 (1.10–1.90)**	2.6
**Subjective peer status**								
High status	371	64.8	202	35.3	1.00		1.00	
Medium status	765	66.0	394	34.0	0.95 (0.77–1.17)	−0.5	0.92 (0.74–1.15)	−0.8
Low status	377	64.9	204	35.1	0.99 (0.78–1.27)	−0.1	0.87 (0.67–1.12)	−1.1
**Subjective scholastic status**								
High status	413	71.2	167	28.8	1.00		1.00	
Medium status	773	66.4	391	33.6	1.25 (1.01–1.55)*	2.0	1.29 (1.04–1.62)*	2.3
Low status	327	57.5	242	42.5	1.83 (1.43–2.34)***	4.8	1.89 (1.46–2.44)***	4.8
**Subjective sports status**								
High status	415	71.4	166	28.6	1.00		1.00	
Medium status	761	66.1	391	33.9	1.28 (1.03–1.60)*	2.3	1.13 (0.91–1.42)	1.1
Low status	337	58.1	243	41.9	1.80 (1.41–2.30)***	4.7	1.48 (1.15–1.92)**	3.0
(*N*)	(1513)		(800)		(2313)		(2313)	

*p < 0.05, **p < 0.01, ***p < 0.001.

**Table 2:** General Health Questionnaire (GHQ-12) ‘case’ according to gender, school year group and status measures: (a) numbers (and row percentages); (b) unadjusted odds ratios (and 95% confidence intervals) showing bivariate associations with gender, year group and each status measure; (c) fully adjusted ORs (and 95% CIs) for model including gender, year group and all status measures.

	(a) GHQ caseness				(b) GHQ case		(c) GHQ case	
	Not a case		GHQ case					
	*N*	row %	*N*	row %	OR (95% CI)	Wald	AOR (95% CI)	Wald
**Gender**								
Males	975	83.1	198	16.7	1.00		1.00	
Females	769	68.0	362	32.0	2.32 (1.90–2.82)***	8.4	2.04 (1.65–2.53)***	6.6
**Year group**								
S2	640	81.3	147	18.7	1.00		1.00	
S3	601	77.6	174	22.5	1.26 (0.99–1.61)	1.8	1.25 (0.96–1.61)	1.7
S4	503	67.8	239	32.2	2.07 (1.63–2.62)***	6.0	2.02 (1.57–2.59)***	5.5
**Residential deprivation**								
High status (low deprivation)	448	79.9	113	20.1	1.00		1.00	
Medium status	680	76.2	213	23.9	1.24 (0.96–1.61)	1.7	1.14 (0.87–1.50)	0.9
Low status (high deprivation)	237	70.8	98	29.2	1.64 (1.20–2.24)**	3.1	1.38 (0.98–1.94)	1.9
Missing	379	73.6	136	26.4	1.42 (1.07–1.89)*	2.4	1.33 (0.98–1.81)	1.8
**Family affluence scale**								
High status (high affluence)	443	76.2	138	23.8	1.00		1.00	
Medium status	924	76.9	278	23.1	0.97 (0.76–1.22)	−0.3	0.91 (0.71–1.17)	−0.7
Low status (low affluence)	377	72.4	144	27.6	1.23 (0.94–1.61)	1.5	0.93 (0.69–1.25)	−0.5
**Subjective SES**								
High status	523	82.7	109	17.3	1.00		1.00	
Medium status	928	76.1	292	23.9	1.51 (1.18–1.93)**	3.3	1.25 (0.97–1.63)	1.7
Low status	293	64.8	159	35.2	2.60 (1.96–3.45)***	6.6	1.75 (1.28–2.39)***	3.5
**Subjective peer status**								
High status	469	81.6	106	18.4	1.00		1.00	
Medium status	900	77.6	260	22.4	1.28 (0.99–1.64)	1.9	1.18 (0.90–1.54)	1.2
Low status	375	65.9	194	34.1	2.29 (1.74–3.01)***	6.0	1.90 (1.41–2.56)***	4.2
**Subjective scholastic status**								
High status	464	80.0	116	20.0	1.00		1.00	
Medium status	897	77.5	260	22.5	1.16 (0.91–1.48)	1.2	1.10 (0.85–1.42)	0.7
Low status	383	67.6	184	32.4	1.92 (1.47–2.51)***	4.8	1.85 (1.38–2.48)***	4.2
**Subjective sports status**								
High status	482	83.3	97	16.7	1.00		1.00	
Medium status	889	77.6	257	22.4	1.44 (1.11–1.86)**	2.8	1.24 (0.95–1.63)	1.6
Low status	373	64.4	206	35.6	2.74 (2.08–3.62)***	7.2	1.99 (1.48–2.68)***	4.5
(*N*)	(1744)		(560)		(2304)		(2304)	

*p < 0.05, **p < 0.01, ***p < 0.001.

**Table 3:** “Get angry when anyone tells me what to do” according to gender, school year group and status measures: (a) numbers (and row percentages); (b) unadjusted odds ratios (and 95% confidence intervals) showing bivariate associations with gender, year group and each status measure; (c) fully adjusted ORs (and 95% CIs) for model including gender, year group and all status measures.

	(a) Anger				(b) Get angry		(c) Get angry	
	Do not get angry		Get angry					
	*N*	row %	*N*	row %	OR (95% CI)	Wald	AOR (95% CI)	Wald
**Gender**								
Males	931	79.6	238	20.4	1.00		1.00	
Females	924	81.7	207	18.3	0.88 (0.71–1.08)	−1.2	1.04 (0.82–1.30)	0.3
**Year group**								
S2	649	83.0	133	17.0	1.00		1.00	
S3	604	78.1	169	21.9	1.37 (1.06–1.76)*	2.4	1.38 (1.06–1.81)*	2.4
S4	602	80.8	143	19.2	1.16 (0.89–1.50)	1.1	1.17 (0.89–1.54)	1.1
**Residential deprivation**								
High status (low deprivation)	478	84.6	87	15.4	1.00		1.00	
Medium status	736	82.0	161	18.0	1.20 (0.90–1.60)	1.3	1.06 (0.79–1.43)	0.4
Low status (high deprivation)	260	78.5	71	21.5	1.50 (1.06–2.12)*	2.3	1.13 (0.78–1.64)	0.6
Missing	381	75.2	126	24.8	1.82 (1.34–2.46)***	3.8	1.35 (0.97–1.87)	1.8
**Family affluence scale**								
High status (high affluence)	487	83.4	97	16.6	1.00		1.00	
Medium status	957	80.3	235	19.7	1.23 (0.95–1.60)	1.6	1.27 (0.96–1.68)	1.7
Low status (low affluence)	411	78.4	113	21.6	1.38 (1.02–1.87)*	2.1	1.23 (0.88–1.71)	1.2
**Subjective SES**								
High status	506	79.3	132	20.7	1.00		1.00	
Medium status	988	81.8	220	18.2	0.85 (0.67–1.09)	−1.3	0.81 (0.63–1.06)	−1.5
Low status	361	79.5	93	20.5	0.99 (0.73–1.33)	−0.1	0.89 (0.63–1.24)	−0.7
**Subjective peer status**								
High status	412	71.7	163	28.3	1.00		1.00	
Medium status	971	84.3	181	15.7	0.47 (0.37–0.60)***	−6.1	0.54 (0.41–0.69)***	−4.8
Low status	472	82.4	101	17.6	0.54 (0.41–0.72)***	−4.3	0.55 (0.40–0.74)***	−3.9
**Subjective scholastic status**								
High status	524	91.0	52	9.0	1.00		1.00	
Medium status	970	83.5	192	16.5	1.99 (1.44–2.76)***	4.2	1.95 (1.40–2.71)***	4.0
Low status	361	64.2	201	35.8	5.61 (4.02–7.83)***	10.2	5.21 (3.69–7.35)***	9.4
**Subjective sports status**								
High status	465	80.9	110	19.1	1.00		1.00	
Medium status	929	80.9	219	19.1	1.00 (0.77–1.29)	−0.0	0.89 (0.68–1.17)	−0.9
Low status	461	79.9	116	20.1	1.06 (0.80–1.42)	0.4	1.01 (0.73–1.39)	0.1
(*N*)	(1855)		(445)		(2300)		(2300)	

*p < 0.05, **p < 0.01, ***p < 0.001.

Focusing first on unadjusted associations with ‘high’ physical symptoms, Table 1, columns ‘a’/’b’ shows these were significantly more likely among females than males, and prevalence increased with age. There were no significant differences by residential deprivation. However, symptoms were *less* likely among those from both mid and low, compared with high family affluence households, and increasingly likely with decreasing subjective SES. Likelihood of ‘high’ physical symptoms also increased with decreasing SSS-scholastic and SSS-sports; SSS-peer was not associated. Table 1, column ‘c’ shows that in the fully adjusted model, ‘high’ physical symptoms remained significantly associated with family affluence (AOR for those ‘low’ vs. ‘high’ status = 0.67), subjective SES (AOR for those ‘low’ vs. ‘high’ status = 1.44), SSS-scholastic (AOR for those ‘low’ vs. ‘high’ status = 1.89) and SSS-sports (AOR for those ‘low’ vs. ‘high’ status = 1.48). The Wald statistics show the most significant effects, apart from those in respect of gender, occurred for SSS-scholastic.

Table 2 shows results for GHQ ‘caseness’. In unadjusted analyses (columns ‘a’/‘b’), levels of ‘caseness’ among females were double those of males, increased with age, and with reductions in status as represented by higher residential deprivation, lower subjective SES and lower SSS-peer, SSS-scholastic and SSS-sports. Family affluence was the only variable not significantly associated with ‘caseness’. In the fully adjusted model (Table 2, column ‘e’), ‘caseness’ was associated with subjective SES and each SSS-school dimension.

Associations with anger (‘I get angry when anyone tells me what to do’) were rather different. Table 3 (columns ‘a’/’b’) shows that in unadjusted analyses there were no gender differences, and rates of anger were highest in the S2 school year (age 14). Anger increased with increasing area deprivation and reducing family affluence, but was the only health/well-being measure not related to subjective SES. Again, contrasting with the other measures, levels of anger were highest among those with *high* SSS-peer. They were also much higher among those with low, compared to high SSS-scholastic. However, anger was not associated with SSS-sports. In the fully adjusted model (column ‘c’), only year group, SSS-peer and SSS-scholastic were associated with anger, with by far the strongest associations in respect of SSS-scholastic (AOR of getting angry when told what to do for those with mid and low vs. high SSS-scholastic were 1.95 and 5.21 respectively).

## 4 Discussion

Our analyses began by comparing levels of subjective SES to those previously reported. US studies have reported mean subjective SES (SSS-society) ranging from 6.4 to 7.2 (Chen and Paterson, 2006, Goodman et al., 2001, Goodman et al., 2007, Goodman et al., 2005), while a Finnish study reported the proportions reporting ‘low’, ‘average’ and ‘high’ (ladder rungs 1–3, 4–7 and 8–10) were 4%, 60% and 36% respectively. Subjective SES among our 13–15 year-old sample was very similar to these results, and decreased with age, also consistent with previous studies (Goodman et al., 2001). Subjective SES was related to family affluence, but not residential deprivation. Other studies have found similarly weak associations between adolescent subjective and objective (particularly neighbourhood-based) SES measures (e.g. Chen and Paterson, 2006). These results indicate that while adolescent subjective SES assessments may be partly based on household/material (but not area-based) characteristics, other factors must also be important (Iversen and Holsen, 2008). Karvonen and Rahkonen (2011) suggest adolescent subjective SES reflects consumerist influences not necessarily aligned with traditional markers of social status like occupation.

Our main aim was to identify the relative importance of objective and subjective SES and the three SSS-school dimensions for adolescent health/well-being. Focusing first on *objective* SES, and consistent with several previous studies, associations with health/well-being were weak or inconsistent, and included higher rates of physical symptoms among more affluent adolescents. Fagg et al. (2013) suggest there may be national differences, with inequalities more likely among US adolescents because of less well-established state-subsidised health and social care systems. Others who have similarly found poorer health among adolescents with higher family affluence note that the scale may represent more than just affluence (e.g. transport requirements, relationships) and that one item, computer ownership, may not have physical health benefits (Koivusilta et al., 2006).

However, *subjective* SES was associated with two of our health/well-being measures (physical symptoms and psychological distress), even after accounting for both objective SES measures. This is consistent with growing numbers of studies of both adults (Adler et al., 2000, Singh-Manoux et al., 2003) and adolescents (Goodman et al., 2007, Karvonen and Rahkonen, 2011) and suggestions that subjective SES represents personal experiences additional to those captured by standard ‘objective’ measures (Singh-Manoux et al., 2003). Interestingly, although objective SES was associated with anger, subjective SES was not.

Importantly, the three SSS-school dimensions showed generally stronger associations with health/well-being than did either objective or subjective SES. This is consistent with others who emphasise the significance of the more immediate (peer/school) social context for adolescent health/well-being (Goodman et al., 2003, Karvonen and Rahkonen, 2011). It is also important from a life-course epidemiology perspective which emphasises the long-term health effects of social factors earlier in life (Kuh et al., 2003). This may be particularly significant for self-reported physical health measures, since the more physiological effects of processes associated with low SSS may not impact immediately.

While further research is required to understand why SSS is linked to health/well-being (Goodman et al., 2001), there are a number of potential mechanisms, particularly in respect of SSS-school. Social exclusion and low classroom status are associated with both physical and psychological health measures (Hertzman and Boyce, 2010, Lynch, 2000). Possessing a stigmatising identity in a context where high and low status groups interact regularly is likely to impact on well-being, and strategies to maintain a more positive identity may take considerable effort (Destin et al., 2012). There is evidence low social status promotes ruminative coping, potentially resulting in poor health outcomes (Jackson et al., 2011). Alternatively, poor health/well-being may prevent (futile) struggles for status (Aslund et al., 2009), raising the issue of direction of causality (discussed later).

One sub-question was whether different SSS-school dimensions were associated with health/well-being in the same way. Among the SSS-school measures, associations were largely consistent with the developmental psychology literature which suggests better health/well-being among those who are more liked, popular, have higher academic interest and achievement and do more sports (Brown et al., 2008, Delsing et al., 2007, Dougherty, 2006, Johnson and Taliaferro, 2011, Koivusilta et al., 2006). However, while SSS-peer was not associated with physical symptoms, our finding of lower psychological distress and greater anger among those with high SSS-peer suggest this represents an amalgam of the two types of popular pupils (liked and visible) which other studies have found overlap (Cillessen and Rose, 2005). It is also consistent with studies which have found that while being liked is positively associated with prosocial/inclusive behaviour, higher status and influence are associated with aggression (Sandstrom and Cillessen, 2006) and, possibly, struggle for status (Eder, 1985, Michell, 1997). Thus, although we did not find SSS-peer to have a U-shaped relationship with any one health/well-being measure, lower psychological distress combined with greater anger for high SSS-peer pupils and greater distress with lower anger for low SSS-peer pupils are consistent with suggestions that longer-term outcomes may be most positive for those at neither extreme on this dimension (Modin et al., 2011). They may also help explain raised cortisol levels previously found among high SSS-peer pupils (West et al., 2010).

Another sub-question asked whether the relative importance of different status measures varied across the three health/well-being measures. We found this to be the case. This variation highlights the need to consider several health/well-being measures, since different relationships might lead to different conclusions about the significance of (different types of) status for health/well-being.

Our final sub-question related to gender differences. Although previous studies (Modin et al., 2011, Oldehinkel et al., 2007, Piko, 2000, Sandstrom and Cillessen, 2006) led us to expect adolescent health/well-being would be more strongly associated with SSS-peer in females and SSS-sports in males, we found no evidence of gender differences in associations between any of the SSS-school dimensions and health/well-being. This is surprising, particularly in respect of SSS-sports, given strong gender differences in adolescent sports participation and interest (Sallis et al., 2000). It appears that even if there are gender differences in *levels* of the SSS-school dimensions, there are none in associations with health/well-being.

Like most similar studies, a significant limitation of our analysis is that it is cross-sectional, meaning we cannot confidently infer direction of causality. Previous studies have found peer status predicts future depression (Almquist, 2009) and that SSS-school remains associated with depression following adjustment for self-esteem (Goodman et al., 2001). There is also evidence that psychological distress may cause low status via behavioural inhibition and withdrawal (Dougherty, 2006) and that health at school is related to subsequent academic attainment (Jackson, 2009). Associations are most likely bi-directional, regardless of either the SSS-school dimensions or the health/well-being measure employed (Dougherty, 2006). However, while the health inequalities literature tends to emphasise relationships *from* status *to* health/well-being, developmental psychology literature emphasises the reverse. A further, related limitation is that our analysis uses self-report data. Self-report is a particular issue for the SSS-school measures. Individuals tend to overestimate positive traits when self-reporting (Aslund et al., 2009), self-reports may be subject to social desirability effects (Delsing et al., 2007), and depressed children may perceive their social status to be more negative than it really is, while aggressive children are less sensitive to social cues (Rudolph and Clark, 2001). Strong inverse relationships between the SSS-school dimensions and psychological distress raise the possibility that these measures reflect nothing more than self-esteem. However, previous analyses found SSS-peer, SSS-scholastic and SSS-sports status each had unique relationships with variables representing more objective and/or self-report behavioural measures (Sweeting et al., 2011); SSS-peer has also been associated with drive, fun- and sensation-seeking (Stautz and Cooper, 2014). There is also evidence that even young children are generally well aware of peers' perceptions of them on behavioural, social status and ability dimensions (Malloy et al., 2007), and that the social status judgements of depressed children are not entirely invalid (Rudolph and Clark, 2001). Further, a meta-analytic review found similar associations between children's social status and negative emotionality *regardless* of type or source of social status measure (Dougherty, 2006). Additional factors, such as parenting style, may also impact on both adolescent SSS-school (Dekovic and Janssens, 1992) and health/well-being (McFarlane et al., 1995). Another limitation is that our measure of physical symptoms, although similar to that used by others (Popay et al., 1993) is a less than ideal indicator of physical health. As numerous authors have pointed out, rates of serious physical morbidity are low in adolescence (Mechanic and Hansell, 1987, Starfield et al., 1993). Biomarker measures such as cortisol (West et al., 2010) would have been the best way to assess the impact of processes associated with low SSS on adolescent physiology, but were not included in our study. Alternative physical health measures include physical fitness, “a powerful marker for health” in children and adolescents (Ortega et al., 2008), (the lengthy) self-report Child Health and Illness Profile (Starfield et al., 1993) which assesses physical discomfort and disorders, or possibly the Short-Form-12 scale (Fonga et al., 2010, Ware et al., 1996). However, previous studies of adolescents have shown physical symptoms such as those included in our analysis to be associated with experience of victimisation, a school/peer-related stressor (Due et al., 2005) and with self-assessed physical health (Mechanic and Hansell, 1987).

Longitudinal studies of subjective social status and health/well-being, including a broader range of self-report and physiological health measures are required, as are investigations of whether SSS-school dimensions are associated with health/well-being in the same ways for adolescents from different socio-economic backgrounds or attending different schools. For example, SSS-scholastic may have stronger relationships with health/well-being in highly academic schools. In addition to ladders measuring subjective societal family (macro-level) SES and separate status dimensions within the school (micro-level) context, further studies might also include the single, overall, SSS-school ladder representing “the people in your school with the most respect, the highest grades and the highest standing” (Goodman et al., 2001) and a ladder or ladders relating to economic status relative to other school-pupils. This would enable comparison of the importance of subjective SES at both macro and micro levels. However, evidence of only weak associations between adolescent subjective SES, more objective measures of family SES and own pocket money (Karvonen and Rahkonen, 2011) suggest measurement of economic status relative to other pupils might need to focus on both family SES and own spending power. Future studies may also increase understandings of how development of social status affects health by giving closer attention to developmental stage. Most research informing our study focused on mid-adolescence, so discerning age-based patterns of association was difficult. Two studies including both SSS-society and SSS-school (Goodman et al., 2001, Page et al., 2009) offer hints that SSS-school may be more important in earlier-mid adolescence when peer acceptance needs may be highest. However, this result could also reflect the different geographical location of the two studies, or other unobserved factors.

In sum, we have shown adolescent self-report health/well-being to have weak and/or inconsistent associations with objective SES, stronger associations with subjective SES and the strongest associations with SSS-school dimensions. Farmer et al. (2011) invokes the idea of the teacher as an ‘invisible hand’, contributing to and facilitating pupils' social interactions and peer relationships and, as such, supporting their social development. Although direction of causality is uncertain, our results, together with suggestions that school-based social status is associated with subsequent social mobility and adult health problems (Modin et al., 2011, Novak et al., 2012, Ostberg and Modin, 2008) highlight the need for a focus on school-based social status among those working to promote adolescent health/well-being.
