# Supplementary material for: Adolescent socio-economic and school-based social status, health and well-being
Source: Soc Sci Med. 2014 Nov;121:39–47. doi: 10.1016/j.socscimed.2014.09.037 (PMC4222198; doi:10.1016/j.socscimed.2014.09.037)
Supplement: Supplementary file 1 [file mmc1.docx]

**SUPPLEMENTARY TABLE 1: Factor analysis (principal components analysis, orthogonal rotation) of school-based subjective social status items – variance explained and rotated component matrix.**

|  |  |  |  |
| --- | --- | --- | --- |
|  | **1** | **2** | **3** |
|  |  |  |  |
| ***% variance explained*** | *50.6* | *17.3* | *9.3* |
|  |  |  |  |
| Powerful | **.844** | .048 | .088 |
| Popular | **.835** | -.019 | .224 |
| Respected | **.775** | .088 | .352 |
| Attractive or stylish | **.772** | -.021 | .291 |
| Doing well at school | .316 | **.844** | .051 |
| Trouble-maker | .511 | **-.693** | .031 |
| Sporty | .306 | .022 | **.933** |
|  |  |  |  |

**SUPPLEMENTARY TABLE 2: ‘High’ physical symptoms, General Health Questionnaire (GHQ-12) ‘case’ and “Get angry ...” - adjusted ORs (and 95% CIs) for modelsincluding gender, year group and all status measures – models based on data weighted for differential attrition and follow-up and clustered within school classes.**

|  |  |  |  |  |  |  |  |  |
| --- | --- | --- | --- | --- | --- | --- | --- | --- |
|  | ***‘High’ physical symptoms*** | |  | ***GHQ-12 ‘case’*** | |  | ***Get angry*** | |
|  |  | |  |  | |  |  | |
|  | **OR (95% CI)** | ***t*** |  | **AOR (95% CI)** | ***t*** |  | **AOR (95% CI)** | ***t*** |
| **Gender** |  |  |  |  |  |  |  |  |
| Males | 1.00 |  |  | 1.00 |  |  | 1.00 |  |
| Females | 1.58 (1.30-1.93)*** | *4.6* |  | 2.09 (1.68-2.60)*** | *6.7* |  | 1.02 (0.83-1.25) | *0.2* |
| **Year group** |  |  |  |  |  |  |  |  |
| S2 | 1.00 |  |  | 1.00 |  |  | 1.00 |  |
| S3 | 1.06 (0.85-1.33) | *0.5* |  | 1.23 (0.90-1.67) | *1.3* |  | 1.40 (1.07-1.83)* | *2.5* |
| S4 | 1.16 (0.93-1.44) | *1.4* |  | 1.98 (1.43-2.73)*** | *4.2* |  | 1.18 (0.91-1.53) | *1.3* |
| **Residential deprivation** |  |  |  |  |  |  |  |  |
| High status (low deprivation) | 1.00 |  |  | 1.00 |  |  | 1.00 |  |
| Medium status | 1.20 (0.94-1.54) | *1.5* |  | 1.15 (0.87-1.54) | *1.0* |  | 1.07 (0.80-1.44) | *0.5* |
| Low status (high deprivation) | 1.08 (0.79-1.47) | *0.5* |  | 1.40 (1.00-1.95) | *2.0* |  | 1.17 (0.80-1.70) | *0.8* |
| Missing | 1.18 (0.91-1.53) | *1.3* |  | 1.32 (0.95-1.82) | *1.7* |  | 1.42 (1.05-1.90)* | *2.3* |
| **Family Affluence Scale** |  |  |  |  |  |  |  |  |
| High status (high affluence) | 1.00 |  |  | 1.00 |  |  | 1.00 |  |
| Medium status | 0.75 (0.58-0.95)* | *-2.4* |  | 0.88 (0.68-1.13) | *-1.0* |  | 1.26 (0.95-1.66) | *1.6* |
| Low status (low affluence) | 0.65 (0.50-0.83)** | *-3.4* |  | 0.91 (0.67-1.23) | *-0.6* |  | 1.23 (0.88-1.71) | *1.2* |
| **Subjective SES** |  |  |  |  |  |  |  |  |
| High status | 1.00 |  |  | 1.00 |  |  | 1.00 |  |
| Medium status | 1.11 (0.89-1.37) | *0.9* |  | 1.20 (0.90-1.59) | *1.3* |  | 0.82 (0.63-1.07) | *-1.5* |
| Low status | 1.47 (1.13-1.92)** | *2.9* |  | 1.63 (1.21-2.20)** | *3.3* |  | 0.89 (0.66-1.20) | *-0.8* |
| **Subjective peer status** |  |  |  |  |  |  |  |  |
| High status | 1.00 |  |  | 1.00 |  |  | 1.00 |  |
| Medium status | 0.91 (0.74-1.12) | *-0.9* |  | 1.13 (0.88-1.46) | *1.0* |  | 0.53 (0.41-0.68)*** | *-4.8* |
| Low status | 0.83 (0.65-1.07) | *-1.5* |  | 1.83 (1.39-2.42)*** | *4.3* |  | 0.53 (0.40-0.71)*** | *-4.3* |
| **Subjective scholastic status** |  |  |  |  |  |  |  |  |
| High status | 1.00 |  |  | 1.00 |  |  | 1.00 |  |
| Medium status | 1.32 (1.03-1.69)* | *2.2* |  | 1.14 (0.87-1.50) | *0.9* |  | 1.97 (1.39-2.80)*** | *3.8* |
| Low status | 1.91 (1.46-2.50)*** | *4.7* |  | 1.90 (1.40-2.57)*** | *4.2* |  | 5.17 (3.63-7.36)*** | *9.2* |
| **Subjective sports status** |  |  |  |  |  |  |  |  |
| High status | 1.00 |  |  | 1.00 |  |  | 1.00 |  |
| Medium status | 1.15 (0.90-1.47) | *1.1* |  | 1.23 (0.92-1.64) | *1.4* |  | 0.92 (0.71-1.19) | *-0.7* |
| Low status | 1.49 (1.14-1.93)** | *3.0* |  | 1.91 (1.39-2.63)*** | *4.0* |  | 1.01 (0.73-1.39) | *-0.1* |
|  |  |  |  |  |  |  |  |  |
| *(N)* | *(2,313)* |  |  | *(2,304)* |  |  | *(2,300)* |  |
|  |  |  |  |  |  |  |  |  |

*p<0.05, *p<0.01, ***p<0.001

**SUPPLEMENTARY TABLE 3: ‘High’ physical symptoms, General Health Questionnaire (GHQ-12) ‘case’ and “Get angry ...” according to year group and status measures: unadjusted odds ratios (OR) with 95% confidence intervals (CI) for males and females and significance of interaction with gender.**

|  |  |  |  |  |  |  |  |  |  |
| --- | --- | --- | --- | --- | --- | --- | --- | --- | --- |
|  | **‘High’ physical symptoms** | | | **GHQ ‘case** | | | **Get angry** | | |
|  |  |  |  |  |  |  |  |  |  |
|  | **Males**  **OR (%% CI)** | **Females**  **OR (95% CI)** | **Signif of gender interactn.** | **Males**  **OR (%% CI)** | **Females**  **OR (95% CI)** | **Signif of gender interactn.** | **Males**  **OR (%% CI)** | **Females**  **OR (95% CI)** | **Signif of gender interactn.** |
| **Year group** |  |  |  |  |  |  |  |  |  |
| S2 | 1.00 | 1.00 |  | 1.00 | 1.00 |  | 1.00 | 1.00 |  |
| S3 | 1.33 (0.98-1.81) | 0.93 (0.69-1.24) | .095 | 1.46 (0.99-2.17) | 1.19 (0.86-1.65) | .433 | 1.42 (0.99-2.01) | 1.30 (0.90-1.87) | .734 |
| S4 | 1.26 (0.92-1.72) | 1.27 (0.95-1.70) | .957 | 1.76 (1.20-2.60) | 2.50 (1.84-3.40) | .169 | 1.11 (0.77-1.59) | 1.21 (0.83-1.76) | .734 |
| **Residential deprivation** |  |  |  |  |  |  |  |  |  |
| High status (low deprivation) | 1.00 | 1.00 |  | 1.00 | 1.00 |  | 1.00 | 1.00 |  |
| Medium status | 1.09 (0.78-1.51) | 1.32 (0.96-1.79) | .414 | 0.84 (0.56-1.27) | 1.53 (1.09-2.14) | .029 | 0.89 (0.60-1.30) | 1.74 (1.13-2.70) | .023 |
| Low status (high deprivation) | 1.21 (0.79-1.86) | 1.07 (0.73-1.58) | .678 | 1.37 (0.83-2.27) | 1.73 (1.15-2.60) | .480 | 1.26 (0.78-2.04) | 1.93 (1.15-3.24) | .238 |
| Missing | 1.11 (0.78-1.59) | 1.42 (0.99-2.04) | .344 | 1.35 (0.88-2.05) | 1.60 (1.08-2.36) | .565 | 1.54 (1.04-2.28) | 2.25 (1.39-3.66) | .229 |
| **Family Affluence Scale** |  |  |  |  |  |  | 1.40 (0.97-2.03) |  |  |
| High status (high affluence) | 1.00 | 1.00 |  | 1.00 | 1.00 |  | 1.00 | 1.00 |  |
| Medium status | 1.11 (0.81-1.52) | 0.61 (0.46-0.81) | .005 | 1.29 (0.87-1.90) | 0.84 (0.62-1.14) | .090 | 1.40 (0.97-2.03) | 1.07 (0.74-1.54) | .307 |
| Low status (low affluence) | 1.03 (0.71-1.50) | 0.64 (0.46-0.90) | .061 | 1.13 (0.71-1.81) | 1.36 (0.96-1.93) | .538 | 1.40 (0.91-2.16) | 1.36 (0.89-2.07) | .911 |
| **Subjective SES** |  |  |  |  |  |  |  |  |  |
| High status | 1.00 | 1.00 |  | 1.00 | 1.00 |  | 1.00 | 1.00 |  |
| Medium status | 0.98 (0.73-1.32) | 1.25 (0.93-1.66) | .254 | 2.02 (1.33-3.05) | 1.24 (0.90-1.69) | .064 | 0.91 (0.66-1.26) | 0.80 (0.56-1.14) | .585 |
| Low status | 1.36 (0.94-1.96) | 1.60 (1.13-2.26) | .527 | 3.15 (1.96-5.06) | 2.24 (1.55-3.22) | .263 | 1.01 (0.67-1.53) | 0.97 (0.63-1.49) | .897 |
| **Subjective peer status** |  |  |  |  |  |  |  |  |  |
| High status | 1.00 | 1.00 |  | 1.00 | 1.00 |  | 1.00 | 1.00 |  |
| Medium status | 0.83 (0.62-1.10) | 0.93 (0.67-1.28) | .601 | 1.13 (0.77-1.64) | 1.10 (0.77-1.57) | .924 | 0.42 (0.30-0.58) | 0.53 (0.37-0.78) | .333 |
| Low status | 0.89 (0.62-1.26) | 0.90 (0.63-1.29) | .947 | 2.43 (1.61-3.68) | 1.65 (1.13-2.41) | .177 | 0.67 (0.46-0.99) | 0.46 (0.30-0.71) | .190 |
| **Subjective schol status** |  |  |  |  |  |  |  |  |  |
| High status | 1.00 | 1.00 |  | 1.00 | 1.00 |  | 1.00 | 1.00 |  |
| Medium status | 1.31 (0.95-1.82) | 1.24 (0.92-1.66) | .791 | 1.44 (0.94-2.20) | 1.08 (0.79-1.48) | .294 | 2.12 (1.34-3.34) | 1.84 (1.15-2.92) | .667 |
| Low status | 1.65 (1.15-2.38) | 2.22 (1.58-3.13) | .244 | 2.10 (1.33-3.29) | 2.14 (1.51-3.04) | .943 | 4.83 (3.02-7.72) | 6.57 (4.09-10.56) | .365 |
| **Subjective sports status** |  |  |  |  |  |  |  |  |  |
| High status | 1.00 | 1.00 |  | 1.00 | 1.00 |  | 1.00 | 1.00 |  |
| Medium status | 1.27 (0.96-1.69) | 1.10 (0.77-1.56) | .526 | 1.37 (0.95-1.98) | 1.14 (0.78-1.67) | .498 | 1.04 (0.75-1.43) | 0.97 (0.63-1.50) | .809 |
| Low status | 1.54 (1.06-2.23) | 1.52 (1.06-2.19) | .971 | 2.41 (1.56-3.73) | 1.95 (1.32-2.89) | .482 | 1.30 (0.86-1.98) | 0.98 (0.62-1.54) | .363 |
|  |  |  |  |  |  |  |  |  |  |
| *(N)* | *(1,178)* | *(1,135)* | *(2,313)* | *(1,173)* | *(1,131)* | *(2,304)* | *(1,169)* | *(1,131)* | *(2,300)* |
|  |  |  |  |  |  |  |  |  |  |
